# Highly tunable β-relaxation enables the tailoring of crystallization in phase-change materials

**DOI:** 10.1038/s41467-022-35005-x

**Published:** 2022-11-29

**Authors:** Yudong Cheng, Qun Yang, Jiangjing Wang, Theodoros Dimitriadis, Mathias Schumacher, Huiru Zhang, Maximilian J. Müller, Narges Amini, Fan Yang, Alexander Schoekel, Julian Pries, Riccardo Mazzarello, Matthias Wuttig, Hai-Bin Yu, Shuai Wei

**Affiliations:** 1 Center for Alloy Innovation and Design (CAID), State Key Laboratory for Mechanical Behavior of Materials, Xi’an Jiaotong University, Xi’an, 710049 China; 2 Institute of Physics (IA), RWTH Aachen University, Aachen, 52056 Germany; 3 Wuhan National High Magnetic Field Center and School of Physics, Huazhong University of Science and Technology, Wuhan, 430074 Hubei China; 4 Department of Chemistry, Aarhus University, 8000 Aarhus, Denmark; 5 Institut für Materialphysik im Weltraum, Deutsches Zentrum für Luft- und Raumfahrt (DLR), 51170 Köln, Germany; 6 Deutsches Elektronen-Synchrotron DESY, 22607 Hamburg, Germany; 7 Department of Physics, Sapienza University of Rome, Rome, 00185 Italy; 8 iMAT Centre for Integrated Materials Research, Aarhus University, 8000 Aarhus, Denmark

**Keywords:** Applied physics, Structure of solids and liquids

## Abstract

In glasses, secondary (β-) relaxations are the predominant source of atomic dynamics. Recently, they have been discovered in covalently bonded glasses, i.e., amorphous phase-change materials (PCMs). However, it is unclear what the mechanism of β-relaxations is in covalent systems and how they are related to crystallization behaviors of PCMs that are crucial properties for non-volatile memories and neuromorphic applications. Here we show direct evidence that crystallization is strongly linked to β-relaxations. We find that the β-relaxation in Ge_15_Sb_85_ possesses a high tunability, which enables a manipulation of crystallization kinetics by an order of magnitude. In-situ synchrotron X-ray scattering, dielectric functions, and ab-initio calculations indicate that the weakened β-relaxation intensity stems from a local reinforcement of Peierls-like distortions, which increases the rigidity of the bonding network and decreases the dynamic heterogeneity. Our findings offer a conceptually new approach to tuning the crystallization of PCMs based on manipulating the β-relaxations.

## Abstract (web summary)

The crystallization of phase-change materials is relevant to their applications in memory and neuromorphic computing devices. Cheng et al. show that the β-relaxation in Ge_15_Sb_85_ can be controlled by thermal annealing, leading to the tunability in its crystallization kinetics by one order of magnitude.

## Introduction

Phase-change materials (PCMs) such as Ge–Sb–Te and Sb-rich alloys can be switched reversibly and rapidly between crystalline and amorphous phases within a few nanoseconds or even sub-nanoseconds by heating through electrical or optical pulses^1–3^. The strong contrast in optical and electrical properties between the switchable states can be used to encode information for data storage^4–6^. By applying a series of electric or laser pulses with proper amplitudes and duration (e.g., iterative RESET or cumulative SET), PCMs manifest the ability of forming a nearly continuous distribution of electrical/optical states. This enables their application in brain-inspired computing hardware such as artificial neurons^7^, phase-change synapses^8,9^, and all-optical neural networks^10^. Researchers have long been pursuing fast switching speed, stable data retention, reduced cycle-to-cycle randomness, and ideal transition-state linearity. For PCM-based devices, all these properties are determined by the crystallization kinetics of PCMs at moderately high temperature and the stability of their amorphous state^11–15^. Yet, these demands inherently compete against each other. For instance, increasing the high-temperature crystallization speed by materials engineering leads to faster switching but typically results in crystallization-prone amorphous phase at lower temperatures, thus undermining data retention. Striking a delicate balance between them requires deeper understanding of the properties of amorphous PCMs and their relation to crystallization propensity.

The properties of amorphous materials have been demonstrated to relate to their relaxation dynamics—the inherent feature of disordered systems^16^. At sufficiently high temperatures, structural rearrangement in a liquid is governed by a single process, whereas dynamics in supercooled liquids split into the primary (α) and secondary (β) relaxations^17^. Below the glass transition temperature *T*_g_, the α-relaxation is frozen-in (i.e., with relaxation time longer than practical experimental timescales), but the β-relaxation is still active in the glassy state, thus becoming the principal source of dynamics in glasses^18^. Due to the close relationship with many important properties, including aging^19^, diffusion^20^, crystallization^21^, stability^22^, and plastic deformation^23^, β-relaxation has been extensively studied in metallic glasses^24,25^, polymers^26^, and molecular glasses^27,28^. The presence of β-relaxation is related to the dynamic heterogeneity^29^ and to the occurrence of fast atomic motion inside weakly connected regions (fast or soft regions), while atoms in other regions are nearly “static”^25,30,31^.

The β-relaxation has been recently detected in amorphous PCMs, manifesting as a prominent excess wing in viscoelastic loss modulus *E*′′ before the α-relaxation peak at a temperature below the glass transition^32^. By contrast, non-PCM chalcogenides (e.g. Ge_15_Te_85_ and GeSe), characterized by slow crystallization kinetics, exhibits vanishingly small β-relaxations despite their similar chemical compositions to PCMs^32^. However, it is unknown whether the β-relaxation plays an important role in crystallization propensity in PCMs. If yes, how can we manipulate the β-relaxation to tune their crystallization behaviors? Here, we show a strong dependency of amorphous stability and crystallization kinetics on the strength of β-relaxations in a PCM. By manipulating the β-relaxation, we demonstrate a counterintuitive effect of thermal annealing on crystallization, which can be rationalized in terms of a diminished local source of atomic dynamics associated with local ordering of spatially heterogeneous structures.

## Results

### Efficient suppression of β-relaxation

**Fig. 1:**
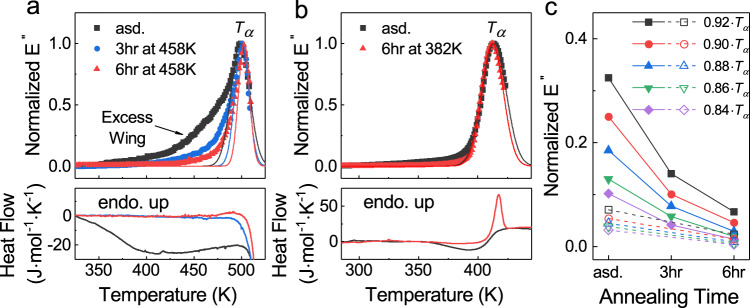
The β-relaxations characterized by powder mechanical spectroscopy (PMS). **a** The normalized loss moduli *E*′′ are measured at 1 Hz for as-deposited (asd.) PCM Ge_15_Sb_85_ and the samples after annealing at 0.92·*T*_*α*_  ≈ 458 K for 3 and 6 h. The as-deposited sample shows a pronounced excess wing in *E*′′, indicating a β-relaxation process. The excess wing is diminished upon annealing. **b** Normalized *E*′′ of as-deposited and annealed non-PCM Ge_15_Te_85_ (annealing at 0.92·*T*_*α*_ ≈ 382 K for 6 h) shows vanishingly small excess wings. Note the temperatures of PMS measurements have been calibrated by subtracting 18 K following the same procedure as in ref. 32. Solid lines in (**a**) and (**b**) represent the Gaussian fits of the main (α-relaxation) peaks. The lower panels of (**a**) and (**b**) show the excess heat flows (*c*_*p*_^*am*^−*c*_*p*_^*xtal*^) of amorphous Ge_15_Sb_85_ and Ge_15_Te_85_ (endothermic up), respectively, measured at 20 K ∙ min^−1^, subjected to the same annealing protocol as in the PMS. **c** The change in *E*′′ intensity with annealing time for amorphous Ge_15_Sb_85_ (solid symbols) and Ge_15_Te_85_ (open symbols) at several temperatures below *T*_*α*_. The uncertainty of the measured normalized *E*′′ is about 2% comparable to the symbol size (see “Methods”).

Ge_15_Sb_85_ is one of the typical Sb-rich alloys of PCMs^33^. It has a higher crystallization temperature of ~520 K at a conventional heating rate, comparing with the conventional PCMs of Ge_2_Sb_2_Te_5_ (~440 K) and GeTe (~480 K), which provides a larger temperature window to investigate and manipulate the relaxation dynamics. We carried out the powder mechanical spectroscopy (PMS) experiment on Ge_15_Sb_85_ to characterize the loss modulus *E*′′ of powder samples^32^. *E*′′ is the viscous component of relaxation spectra and is a measure of dissipated energy, which has been shown as an effective indicator of relaxation events^25^. Figure 1a shows the measured temperature-dependent *E*′′ of the as-deposited Ge_15_Sb_85_ powder sample at 1 Hz. The main peak in *E*′′-curve represents the α-relaxation process with the peak temperature *T*_*α*_ ~ 500 K, which is associated with (de)vitrification, as seen in all known glass systems. Meanwhile, the *E*′′-curve shows an asymmetric feature with a pronounced excess wing at the left flank of the α-relaxation peak. This excess wing indicates the presence of a β-relaxation in the as-deposited Ge_15_Sb_85_. Excess wings are a typical feature of β-relaxations commonly observed in many polymers, molecular, and metallic glasses, where the timescales of α- and β-relaxations decouple to a moderate extent^25^. The lower panel shows the excess heat flow measured using Differential Scanning Calorimetry (DSC) (“Methods”). The exotherm dip indicates an enthalpy release upon heating and no distinguishable calorimetric glass transition is observed before crystallization sets in.

As a reference, we conducted the PMS experiment on as-deposited Ge_15_Te_85_, a non-PCMs with similar chemical composition as Ge_15_Sb_85_ (except Sb is substituted by Te). Unlike Ge_15_Sb_85_, Ge_15_Te_85_ is characterized by good glass-forming ability and slow crystallization kinetics^34^. In Fig. 1b, the PMS measurement shows that the as-deposited Ge_15_Te_85_ exhibits a steady *E*′′ below the glass transition and then a rather symmetric α-relaxation peak centered at *T*_*α*_ ~ 415 K, with a vanishingly small excess wing (β-relaxation) prior to the primary relaxation.

The strength of β-relaxation can be modified by thermal treatment at the temperature below *T*_*α*_, as demonstrated in many glassy systems such as metallic glasses^24,25^. We annealed the as-deposited Ge_15_Sb_85_ sample at 0.92·*T*_*α*_ ≈ 458 K isothermally for 3 h and 6 h. At this chosen temperature, the magnitude of excess wing is high while the contribution from the α-relaxation peak is minimized (Fig. 1a). The *E*′′ of the annealed Ge_15_Sb_85_ was measured and compared to the as-deposited sample. As shown in Fig. 1a, both the magnitude and width of the excess wing decrease substantially upon annealing, and the 6 h annealing leads to a further reduction of the excess wing region than that of 3 h. It is not feasible to determine the accurate peak position of β-relaxation due to the strong overlap with the α-relaxation peak, but we see a clear trend for β-relaxation (the excess wing) to merge into the α-relaxation after annealing of Ge_15_Sb_85_. To make a quantitative comparison, we characterized the suppression of the β-relaxation by extracting the normalized *E*′′-intensities at various temperatures below *T*_α_(see Fig. 1c). In Ge_15_Sb_85_, the strength of β-relaxation is reduced by ~60% with 3 h annealing, and then by a further 20% with the following 3 h, showing considerably large tunability. By contrast, Ge_15_Te_85_ shows almost unchanged shape of the mechanical relaxation spectrum after annealing with a similar protocol at 0.92·*T*_α_ ≈ 382 K for 6 h (Fig. 1b, c), indicating the very limited influence of thermal annealing on relaxation dynamics. The difference between Ge_15_Sb_85_ and Ge_15_Te_85_ has also manifested in their enthalpy relaxations. The former has an enthalpy release of ~3.17 kJ/mol for 3 h and ~3.32 kJ/mol for 6 h with respect to the as-deposited sample; yet, in the latter a five times smaller enthalpy release (~0.65 kJ/mol) is observed upon 6 h annealing.

We note that the suppression of β-relaxation through thermal annealing in Ge_15_Sb_85_ is surprisingly effective and the change in magnitude is rather large compared to most of other types of glasses. For instance, the metallic glass La_60_Ni_15_Al_25_ requires ~125 h of annealing at ~0.94*T*_*g*_^35^ and the polyvinylethylene requires 11 days at ~0.94*T*_*g*_^36^, to reduce the *E*′′ peak intensity of β-relaxations by ~10–20%. An earlier study estimated that most glasses would require thousands to millions of years of annealing to suppress the intensity of β-relaxation by a factor of 3 (ref. 37). The suppression in our case is even more efficient than that of forming ultrastable glasses, in which only 30% suppression of β-relaxation was achievable^37^.

### The counterintuitive effect of annealing on crystallization

Although thermal annealing may have a complex effect on crystallization behaviors in different types of glasses, for those poor glass-forming PCMs it has been often suggested that thermal annealing would facilitate crystallization, as it may introduce more (subcritical) nuclei or growth of nuclei in the glasses, which has been demonstrated in refs. 38–40. In the following, we show that the annealing can introduce a counter-effect, i.e. suppression of β-relaxations, which effectively slows down crystallization kinetics and stabilizes the amorphous phase, and becomes a dominant factor in crystallization.

**Fig. 2:**
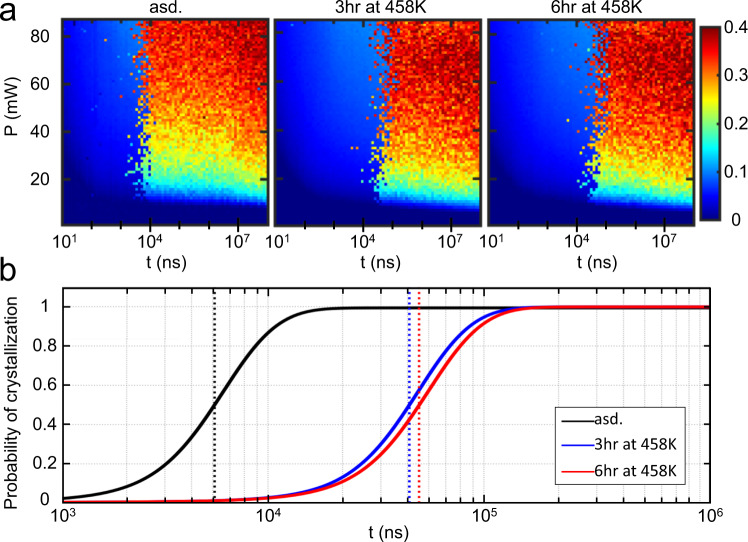
Crystallization time as a function of laser pulse duration for the as-deposited, 3 and 6 h annealed Ge_15_Sb_85_ films. **a** Power-time-effects (PTE) diagrams of the as-deposited, 3 and 6 h annealed Ge_15_Sb_85_ thin films. The *x*-axis and *y*-axis represent the pulse duration *t* (ns) and power *P* (mW), respectively. The reflectance contrast *ΔR* is encoded in the color bar. **b** The probability of crystallization as a function of pulse duration during a fine pulse duration scan at a constant power of ~40 mW. The fitted curves are based on the Gompertz function, and the standard deviation for the as-deposited, annealed at 458 K for 3 h, and for 6 h films are 3.52, 1.85, and 3.51%, respectively (Supplementary Fig. 3). The vertical lines represent the deduced characteristic time *t*_min_. The pre-annealing increases the *t*_min_ of Ge_15_Sb_85_ by around one order of magnitude.

We measured the crystallization rates of amorphous Ge_15_Sb_85_ thin film samples (50 nm in thickness) with a pump-probe laser setup, including an as-deposited and two annealed films with the same thermal protocol as for the PMS experiments. The thin films were exposed to laser pulses with varying power range from 10 to 85 mW, and the duration range from 10 to 10^8^ ns at different spots. The onset of crystallization can be determined by measuring the reflectance contrast *ΔR* before and after each pulse, since the crystallization would induce a dramatic increase of reflectance^41^. Figure 2a depicts the power-time-effect (PTE) diagrams of as-deposited and annealed Ge_15_Sb_85_ films, in which the *ΔR* were recorded as a function of the pump laser power (*P*) and the pulse duration (*t*). A strong optical reflectance contrast can be observed upon a relatively high and long laser pulse, which is induced by the crystallization of amorphous films^42^. The observed highest contrast *ΔR* of 35–40% is consistent with the reflectivity change of typical PCMs upon crystallization determined by stationary reflectivity^41^. The borders between amorphous and crystallization regions in the PTE diagrams correspond to the minimum crystallization time (*t*_min_) of the films. Strikingly, the border shifts to longer timescales after the thermal annealing of 3 and 6 h.

The *t*_min_ is approximately independent of laser power above ~20 mW. Thus, we determined the exact *t*_min_ with the fine pulse duration scans at a constant power of ~40 mW. In a narrower timescale range of 10^3^–10^6^ ns, we performed the laser measurements with 30 different pulse durations, and for each pulse duration, 30 pump pulses were applied at different areas of the sample (see details in Methods and also in ref. 42). The minimum crystallization time *t*_min_ and the probability of crystallization of each pulse duration can be extracted by fitting the data with the Gompertz function (Supplementary Fig. 3). As shown in Fig. 2b, the vertical lines in each fitted curve represent the deduced *t*_min_ of different films. The *t*_min_ of the as-deposited film is ~5.1 × 10^3^ ns, while the value is increased by around an order of magnitude for 3 h annealed film (~4.1 × 10^4^ ns), and further extended to ~4.5 × 10^4^ ns for 6 h annealed one. Note that the *t*_min_ measured here includes the incubation time of crystal formation, which leads to larger values of minimum crystallization time than that in the actual PCM devices^43,44^. In addition, suppressing the β-relaxation has extended the time span *Δt* between 5% and 95% probability of crystallization by a factor of 8 from ~10,000 ns (as-deposited) to ~80,000 ns (3 and 6 h annealed) (Fig. 2b). Such a larger time span reveals a more gradual change in crystallization probability with pulse duration. This might be interesting for applying PCMs for error-tolerant stochastic computing devices^45^ (a similar concept was demonstrated in metal-filament-based memristive devices^46^) because a larger graduality allows the access to a larger number of probability states of crystallization with a higher accuracy.

**Fig. 3:**
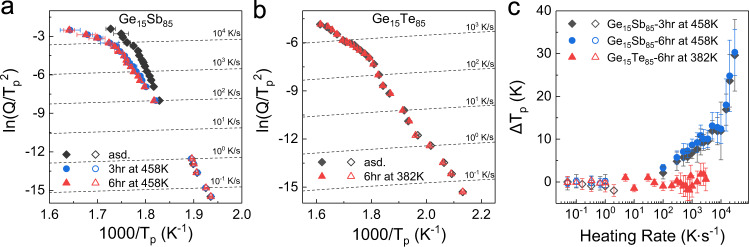
Kissinger plots for the crystallization processes of Ge_15_Sb_85_ and Ge_15_Te_85_. The crystallization peak temperatures *T*_*p*_ at low and high heating rates *Q* are measured by DSC (open symbols) and ultrafast Flash DSC (solid symbols), respectively. **a** Crystallization of as-deposited Ge_15_Sb_85_, and the samples annealed for 3 and 6 h at 458 K. The error bars are the standard deviations of averaging ten times of measurements (see “Methods” and Supplementary Table 1). **b** Crystallization of as-deposited Ge_15_Te_85_ and the sample annealed for 6 h at 382 K. The error bars are estimated less than 0.3% within the size of the symbols. **c** The difference in *T*_*p*_ between the annealed and as-deposited samples of Ge_15_Sb_85_ and Ge_15_Te_85_ for different heating rates *Q*. The error bars are calculated based on the error propagation from (**a**) and (**b**).

We also applied the conventional DSC and ultrafast Flash DSC (FDSC) to further characterize the crystallization kinetics of the samples at different heating rates *Q* ranging from 0.05 K/s to 30,000 K/s (see details in “Methods”). We extracted the temperature of the principal crystallization peak *T*_*p*_ as a function of *Q* and presented it in a Kissinger plot^47,48^. Earlier studies showed that *T*_*p*_ corresponds to a fixed fraction of crystallinity of approximately 63%^47^. Under a given *Q*, the higher *T*_*p*_ indicates the better stability of the amorphous state and slower crystallization. As shown in Fig. 3a, b, *T*_*p*_ of Ge_15_Sb_85_ is clearly shifted to higher temperatures after thermal annealing, while *T*_*p*_ of Ge_15_Te_85_ shows nearly overlapping data points before and after thermal annealing. Figure 3c shows the temperature differences of *T*_*p*_ between the as-deposited and annealed samples, *ΔT*_*p*_ = *T*_*p,*annealed_ *− T*_*p,*asd_. For Ge_15_Sb_85_, the *ΔT*_*p*_ is evident when *Q* > 100 K s^−1^. This is approximately the same heating rate, where the *Q*-dependent *T*_*p*_ crosses the estimated characteristic temperature of β-relaxation, *T*_*β*_ (see Supplementary Fig. 24). The latter is the temperature where the intensity of β-relaxation reaches the maximum value at a given frequency (or inverse timescale). This implies that the β-relaxation starts to dominate the crystallization when the timescales of the two processes are comparable. *ΔT*_*p*_ becomes increasingly larger up to ~30 K with higher heating rates. The change in *ΔT*_*p*_ is more pronounced for the first 3 h annealing than for the subsequent hours, analogous to the change in intensity of β-relaxation in Fig. 1a and *t*_min_ in Fig. 2b. By contrast, the *ΔT*_*p*_ of Ge_15_Te_85_ is virtually zero at all heating rates, indicating the nearly identical crystallization kinetics.

The counterintuitive trends in *t*_min_, *T*_*p*_, and *ΔT*_*p*_ in Ge_15_Sb_85_ suggest that thermal annealing has introduced an opposite effect that dominates the crystallization process. Given that β-relaxation is associated with atomic motion in some local regions that is substantially faster than that in other regions^16,49^, those local fast atoms may contribute to a higher kinetic factor of both nucleation rate *I*_*s*_ and crystal growth *u*^50^. Thus, the slowdown of crystallization can be attributed to the inhibited local mobility of atoms from the thermal annealing, which might also be the origin of increased apparent viscosity argued in other studies^51^. This attribution is supported by a recent computational study by Dragoni et al., where the enhanced stability of ultrathin (3–5 nm) amorphous films of Sb is attributed to the lack of β-relaxations resulting from the nanoconfinement^52^.

### Local reinforcement of Peierls-like distortion

**Fig. 4:**
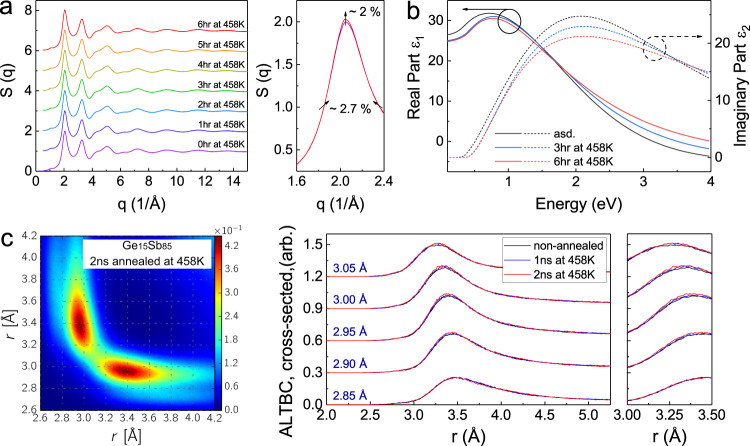
The structural and bonding changes during the isothermal annealing of the amorphous Ge_15_Sb_85_ at ~458 K. **a** The *S(q)* patterns during the isothermal annealing at ~458 K for 6 h. The spectra are vertically shifted for an easy view. The right panel shows the zoom-in of the first peak, where an increase in peak intensity and a narrowing of the full-width-half-maximum (FWHM) are observed upon annealing. Note only the 0 h-annealed and 6hr-annealed curves are shown here. More *S(q)* data within the time interval and the difference *ΔS(q)* are shown in Supplementary Fig. 9 and the uncertainty analysis is given in the “Methods” section. **b** Dielectric functions for amorphous as-deposited and annealed (at ~458 K) Ge_15_Sb_85_ thin films (see “Methods” for an error analysis). The solid lines and dashed lines are the real part and the imaginary part, respectively. **c** The analysis of the angular limited three-body correlation functions (ALTBC) obtained from the AIMD simulations. The left panel shows the 2D contour plot for the total ALTBC at ~285 K after annealing at ~458 K for 2 ns. The cross-sectioned ALTBC (center panel and right panel) shows an increase in peak height upon annealing at ~458 K for 1 ns and 2 ns for given short bond lengths (*r* = 2.85–3.05 Å), indicating reinforcement of Peierls-like distortions.

To understand the structural origin underlying the β-relaxation, we performed the in situ synchrotron X-ray scattering experiment at DESY P02.1 beamline to monitor the structural changes during the isothermal annealing of 6 h at 458 K. After necessary corrections and normalization, the structure factors *S(q)* were extracted from the diffraction patterns and Fourier transformed into the reduced pair distribution functions *G(r)* (“Methods” and Supplementary Fig. 10). No sharp Bragg peaks can be observed in *S(q)*, indicating that the sample retains its amorphous phase during the entire annealing process and no crystallization occurs (Fig. 4a). The zoom-in panel of the first peak of *S(q)* reveals a non-negligible increase in peak intensity and a narrowing of width, implying the structural ordering process and a more rigid atomic packing. Note that the diffraction patterns provide the information of averaged structural changes over the entire scattering volume (~10^5^ μm^3^). Considering that the local ordering related to the suppression of β-relaxation may contribute only a small fraction of the scattering volume, it is probably not surprising that the changes in overall signals in *S(q)* and *G(r)* after averaging appear rather modest.

The dielectric function is a rather sensitive measure of atomic arrangement. Hence, we have performed measurements of the dielectric function, a bond-alignment sensitive indicator^53^, to investigate the underlying mechanism of the structural ordering process. Using Fourier transform infrared spectroscopy (FTIR), we measured the optical reflectance of Ge_15_Sb_85_ films and determined the real (*ε*_1_) and imaginary (*ε*_2_) parts of dielectric functions (“Methods” and Supplementary Fig. 23). In Fig. 4b, a continuous decrease in the maximum height of *ε*_2_ can be observed upon thermal annealing. In *p-*bonded systems with octahedral arrangement, perfect *p*-alignment would result in a maximum overlap of the wave functions, i.e., a large matrix element for optical excitation, and thus a larger value of dielectric function^54,55^. Hence, the decreased *ε*_2_ in the annealed Ge_15_Sb_85_ indicates an overall enhanced distortion of *p*-bonded local structures, i.e. Peierls-like distortion, where the alternating short and long bonds forming on the opposite sides of a central atom lower the total energy of the system. Such an enhancement may come from two sources (1) the enhanced distortion primarily in the regions of β-relaxation and (2) the higher distortion across the entire sample (i.e. homogenous ordering). Clarifying the dominant mechanism requires atomic-scale insight from simulations.

We conducted ab-initio molecular dynamics (AIMD) simulations on annealing of amorphous Ge_15_Sb_85_ (“Methods” and Supplementary Figs. 11–22). The model includes 1080 atoms with the dimension of 32.283 Å × 32.283 Å × 32.283 Å and the corresponding number density of 0.0321 1/Å^3^. We employ the angular limited three-body correlation function (ALTBC) for further structural analysis. This quantity describes the probability of having a bond of a given length *r*_1_ almost aligned with a bond of length *r*_2_. We include angular deviations smaller than 25° in the calculation of this probability. The ALTBC contour plot displays two peaks that correspond to alternating short and long bonds (Fig. 4c, left panel and Supplementary Figs. 13–16), indicating the presence of Peierls-like distortions^56^. After annealing for one million AIMD steps (i.e. 2 ns in total), the peak height of the total cross-sectioned ALTBC in Fig. 4c (right panel) increases (dominated by the Sb-centered motifs, as evident from the partial ALTBC in Supplementary Figs. 15, 16 and also the Sb-centered nearest neighbor distribution in Supplementary Figs. 17–21), while the shift of the peak position is negligible. The former indicates that more atoms (likely from the β-relaxation regions) are involved in Peierls-like distortions upon annealing, while the latter reveals that the existing Peierls-like distortions do not become more pronounced. We denote such a heterogeneous structural change as the local “reinforcement” of Peierls-like distortions, which differs from the scenario of homogeneous ordering. It is consistent with the more rigid atomic packing observed in diffraction patterns and may explain the weakened signals in *ε*_2_ maximum. Hence, both AIMD and experimental results suggest that a heterogeneous structural ordering and the shrinkage of the loosely bonded regions are the underlying mechanism of weakening of the β-relaxations, leading to the structure and bonding with a higher degree of rigidity and covalency like those in non-PCM chalcogenide glasses with conventional covalent bonding. A higher covalency was shown to correlate with the weaker β-relaxations in amorphous GeTe–GeSe alloys with a higher GeSe-content^32^. The latter has been associated with a decreasing crystallization propensity^42^. Recent studies highlighted the difference in properties and bonding between crystalline PCMs (e.g. Ge_15_Sb_85_, GeTe) and non-PCMs (e.g. Ge_15_Te_85_, and GeSe)^57^. The present study reveals a clear difference in relaxation behaviors and crystallization in amorphous states of the two groups.

## Discussion

We have identified the highly tunable β-relaxation in the PCM Ge_15_Sb_85_ and shown that thermal annealing below *T*_g_ can effectively suppress the strength of β-relaxation. Both pump-probe laser and (F)DSC measurements revealed that the suppression of β-relaxation played a dominant role in the drastic slowdown of crystallization and a clear enhancement of amorphous stability. This can be attributed to the diminished source of atomic dynamics (i.e. local fast atomic motion) upon reducing the local disordered regions corresponding to the β-relaxation through a mechanism of reinforcement of the Peierls-like distortion. The good tunability of the β-relaxation in Ge_15_Sb_85_ implies that the bonding, structural and dynamic spatial heterogeneities are sensitive to thermal treatments in this special kind of covalent glasses. Such properties are not observed in the non-PCM Ge_15_Te_85_ despite their similarity in chemical compositions. At last, we note that modification of β-relaxation strength can be achieved by other methods such as varying processing conditions^37^, composition^58,59^, temperature, pressure^60,61^, and strain. A study of Pd-based metallic glasses showed that when the ultrasonic vibrations are stochastically resonant with the atomic motion associated with β-relaxations, crystallization can be accelerated by nearly one order of magnitude^62,63^. The manipulation of β-relaxation provides a conceptually new approach to tailor the crystallization kinetics and the stability of the amorphous phase in PCMs, which is potentially beneficial for their applications in nonvolatile phase-change memory and neuromorphic devices.

## Methods

### Sample preparations

Powder samples of amorphous PCMs were prepared via DC magnetron sputtering (a base pressure of 2 × 10^−6^ mbar and an argon gas flow of 20 sccm) from stoichiometric targets with purity higher than 99.99%. The deposition time was adjusted to yield a film with thickness of ~5 µm. The as-deposited film was scraped off from the substrates afterward for FDSC/DSC/PMS and X-ray scattering measurements.

### PMS measurements

The exfoliated flakes were carefully milled into powders to perform PMS analysis. The mechanical relaxations were studied using the TA Q800 dynamical mechanical analyzer (DMA) together with a custom-designed powder container. Please refer to ref. 32 for more experimental details. Since the absolute values of PMS measurements depend on the amount (e.g., mass) and state (e.g., compactness) of powder sample, the absolute values carry no physical meanings for the intrinsic properties of the materials. Only the relative changes in *E*′′ spectra are presented for comparing the relaxation behaviors. Nonetheless, we were careful to keep the same amount (~180 mg, which is the optimal amount of sample based on our long-term PMS measurement experience) and the same state (e.g., compactness, which is closely related to the compaction state of powder samples by the clamp when the coarse particle size of powder samples is the same) of powder samples for each measurement for the data reliability. The torsional force applied to the powder clamp was maintained at 5 pounds, which ensured that the powder compactness was the same in each PMS measurement. By combining all sources of error and available empirical optimization, we estimate an error of less than 2% in the measured normalized *E*′′ curve (Fig. 1). Pre-annealed powder samples were obtained by in situ annealing inside DMA chamber under Ar. The whole spectra including both *E*′ and *E*′′ can be found in Supplementary Figs. 1, 2). Note that all the temperatures of PMS results have been calibrated by subtracting 18 K, using the same method as in ref. 32.

### Pump-probe laser reflectivity measurements

To perform in situ pump-probe reflectivity measurements, ~50 nm Ge_15_Sb_85_ thin films on silicon substrate are sandwiched between two layers of ZnS–SiO_2_ (prepared via RF sputtering). After the deposition, thermal annealing has been performed at 458 K in a glass tube with a heating rate of 5 K/min and various holding times under Ar atmosphere. In situ reflectivity measurements during phase transformation were performed with a static optical tester. Firstly, to crystallize the thin amorphous phase-change film, a focused laser pulse (wavelength = 658 nm) with variable power and duration is applied to a submicron region. Then, in situ reflectivity is recorded by a low-intensity continuous-wave laser (wavelength = 639 nm) at the same position with the pump laser. The relative change in reflectance is *ΔR* = *(R*_*i*_ *−* *R*_*f*_*)/R*_*i*_ where *R*_*i*_ and *R*_*f*_ are initial and final reflectivity, respectively. We tested the replicability of the reflectivity results (Fig. 2a) by measuring a separate set of samples. The two independent measurements show an excellent agreement (Supplementary Fig. 4).

### DSC and FDSC

For Ge_15_Sb_85_, conventional differential scanning calorimetry (DSC) measurements were performed in a PerkinElmer Diamond DSC for heating rates ranging from 0.05 to 1 K/s. Since the sample mass for the conventional DSC is on the order of 10 mg, a high heating rate well above 100 K/min might cause a thermal lag that might undermine the accuracy of the DSC measurement. Thus, we use the heating rates no larger than 60 K/min (i.e. 1 K/s). The Ultrafast DSC (or FDSC) measurements were performed using the Mettler–Toledo Flash DSC1 for high heating rates from 100 to 30,000 K/s). A too small heating rate (below 100 K/s) often leads to much higher noise levels (Supplementary Fig. 6), because at a low heating rate, the heat flux is lower (i.e. the power measured in [W] is lower). The signal that is picked up by the sensor is lower, resulting in a poorer signal-to-noise ratio. This is why there is a heating-rate gap between the two techniques for Ge_15_Sb_85_. However, we performed the temperature calibration for each heating rate with respect to the standard melting point of indium for the two techniques. This ensures that the measured *T*_*p*_ values in different temperature regimes using the two techniques are reliable (±1 K). Ten scans for each heating rate were carried out to ensure sufficient statistics in the flash DSC measurements and the averaged *T*_*p*_ values are obtained with the standard deviations tabulated in Supplementary Table 1. For Ge_15_Te_85_, the measurement was performed using a Mettler–Toledo DSC 3 combined with the 2nd generation flash DSC model, MT Flash DSC 2+, which, with technical improvements, covers a larger rate range, leaving no heating-rate gap in Fig. 3b. Note that a *Q* > 3000 K/s, partially bypassing the crystallization, shifts *T*_*p*_ into the melt of Ge_15_Te_85_ due to its low melting point (385 °C). Thus, crystallization temperature can be only measured up to *Q* ~ 3000 K/s for Ge_15_Te_85_. The complete DSC/FDSC scans can be found in Supplementary Figs. 5–8.

### In-situ synchrotron X-ray scattering

In situ synchrotron X-ray scattering experiments were performed at DESY Hamburg, PETRAIII, P02.1. The X-ray beam size was 0.5 × 0.5 mm^2^ with a wavelength of 0.207 Å. The as-deposited powder sample of Ge_15_Sb_85_ was loaded and sealed in a silica quartz capillary with an inner diameter of 1 mm. The sample was heated from 300 to 458 K at the same rate as in PMS (3 K/min) and held at 458 K isothermally for 6 h using an Oxford Cryostream furnace. The diffraction patterns were collected in situ in a transmission mode with an exposure time of 10 s using a Varex XRD 4343CT detector (2800 pixels × 2800 pixels, 150 × 150 μm^2^ pixel size). The obtained two- dimensional raw X-ray diffraction patterns were integrated to obtain the intensity *I(q)* using the DAWN data analysis software^64^. The *I(q)* data were processed using PDFgetX2^65^ to correct for background, sample absorption (assuming a constant attenuation coefficient) and multiple scattering (2nd only). Then fluorescence and Compton scattering corrections were applied with the parameters that were refined in an optimization process described in ref. 66. The corrected *I’(q)* was used to calculate the total structure factor $S\left(q\right)=\frac{{I}^{\prime}\left(q\right)}{{\left\langle f(q)\right\rangle }^{2}}+\frac{{\left\langle f(q)\right\rangle }^{2}-\left\langle {f}^{2}(q)\right\rangle }{{\left\langle f(q)\right\rangle }^{2}}$ and its Fourier transform, reduced pair distribution function $G\left(r\right)=\frac{2}{\pi }{\int }_{0}^{\infty }q[S\left(q\right)-1]\sin ({qr}){{{{{{\rm{d}}}}}}q}$, where *f(q)* is the atomic form-factor defined in ref. 67 (*p*.158) and <…> is the compositional average of the form-factors of constituent atomic species tabulated in the literature^67^. The *q*-resolution is 0.005 Å^−1^ for our experimental set-up (i.e. quarter-ring data collections on the detector of 2800 pixels × 2800 pixels, 150 × 150 μm^2^ pixel size, sample-to-detector distance 749.515 mm), which corresponds to an uncertain of ~0.25% in the *q*-region of the first main *S(q)* peak (~2 Å^−1^). The uncertainty of the relative diffracted intensity is reflected in the scattering of the data points in the *S(q)* vs *q* plots. For each diffraction pattern, we integrated the data over 10 s. The error for the integrated intensity is calculated to be about 0.03% (at *q* ~ 2 Å^−1^) using pyPAI (assuming a Poisson error model). The scattering of data points of the integrated intensities is as small as almost invisible in the plot (Supplementary Fig. 9a). Since what matters the most is the relative changes of *S(q)*, instead of the absolute values of *S(q)*, we keep all experimental conditions the same during the entire measurement so that their changes in the *S(q)* peaks can be monitored. The Fourier transform to *G(r)* may introduce additional errors because of the truncation effects of *S(q)* at the maximum *q*-value, *q*_max_. Earlier studies showed that if *q*_max_ is equal or smaller than 5 Å^−1^, the truncation effects on the first and second peaks become significant^66^. In the present study, the *q*_max_ is taken at about 14 Å^−1^. Thus, the truncation effect on the first two peaks of *G(r)* is considered to be negligible.

### Fourier transform infrared spectroscopy (FTIR)

To perform FTIR measurements, Ge_15_Sb_85_ thin films with thickness of ~650 nm were deposited on Si 〈100〉 substrates using same sputtering parameters as above. The reflectance spectra were measured with a Bruker VERTEX 70 spectrometer in the range from 400 to 8000 cm^−1^, with a resolution of 4 cm^−1^. Both as-deposited and annealed samples were measured at room temperature. Besides, the samples are placed in vacuum below 10^−5^ mbar to reduce additional light absorption by gases. Reflectance spectra of as-deposited and annealed samples were measured subsequently to exclude drift effects. Every spectrum results from averaging of 64 scans to obtain the best signal to noise ratio. The relative measurement error for the reflectance is ~0.2% in the measured wavelength range. For every sample, five thickness values were taken at different cross-section positions via scanning electron microscope, and the error was estimated to be within 2%. The average thickness values were used as a reference for the optical simulations. Experimentally obtained reflectance spectra were fitted using the CODE software. The dielectric model function *ε*(ω) consists of the two parts: (1) a constant real part background accounting for the polarizability of the higher energy range and (2) a Tauc–Lorentz model to describe inter-band transitions. A Downhill simplex method was used for the unconstrained optimization. All the parameters were fitted to convergence with variation less than 0.5%.

### AIMD simulations

Ab initio molecular dynamics (AIMD) simulations of Ge_15_Sb_85_ based on density functional theory (DFT) were carried out with the second generation Car-Parrinello-like MD scheme developed by Kuehne et al.^68,69^ and implemented in the CP2K package^68,69^. Two minimization steps were performed for each MD step. We employed the friction coefficients *γ*_L_ = 4.0 × 10^−4^ fs^−1^ and *γ*_D_ = 3.5 × 10^−5^ fs^−1^ (where *γ*_D_ corresponds to the intrinsic dissipation^68,69^, whereas *γ*_L_ is the coefficient of the Langevin thermostat). The Kohn–Sham wave functions were expanded in a triple-zeta plus polarization Gaussian-type basis set, and the charge density was expanded in plane waves with a cutoff 300 Ry. Scalar-relativistic Goedecker pseudopotentials and the standard exchange correlation potential parameterized by Perdew, Bruke, and Ernzerhof (PBE) were used^70,71^. The Brillouin zone was sampled at the Γ point. 1080 atoms were placed in a cell with dimension of 32.283 Å × 32.283 Å × 32.283 Å, and the corresponding number density was: 0.0321 Å^−3^ (ref. 72). The system was firstly randomized for 30 ps at 3000 K, followed by a subsequent equilibration for 30 ps at 1335 K. The melt-quenching model was obtained by quenching (3 K/ps) to 300 K. Note that this quenching rate is still rather faster than the experimental values. Re-annealed samples were obtained by equilibration at/re-heating to target temperatures (~458 K) for 500 k MD steps = 1 ns and 1 M MD steps = 2 ns. Finally, the system was re-quenched to ~285 K and equilibrated for 30 ps before further analysis. We computed the pair distribution functions *g(r)*, and the structure factor *S(q)* was calculated from the partial distribution function by means of Fourier transform. The angular limited three-body correlation (ALTBC), which describes the probability of having a bond length *r*_*1*_ almost aligned with a bond length *r*_*2*_, was also computed from the AIMD trajectories (see Supplementary Fig. 13). More details can be found in ref. 72. We note that the increase in Peierls-like distortions is typically accompanied by increase in volume^73^. However, combining the efficient 2nd-generation Car-Parrinello scheme with constant-pressure simulations poses technical challenges. Thus, AIMD was carried out with a constant-volume protocol. The fact that we do observe a sharpening of the ALTBC peaks in spite of the constant-volume “constraint” indicates that the reinforcement of the Peierls-like distortions is a genuine effect.

## Supplementary information

Supplementary Information

## Data Availability

The authors declare that all data supporting the finding of this study are available within the paper.

## Code availability

The AIMD trajectories will be available from the corresponding author upon request.
