# Supplementary material for: Highly tunable β-relaxation enables the tailoring of crystallization in phase-change materials
Source: Nat Commun. 2022 Nov 29;13:7352. doi: 10.1038/s41467-022-35005-x (PMC9709056; doi:10.1038/s41467-022-35005-x)
Supplement: Supplementary file 1 — Supplementary Information [file 41467_2022_35005_MOESM1_ESM.pdf]

Supplementary Information

Highly tunable β-relaxation enables the tailoring of crystallization in phase-change materials

Yudong Cheng[1,2†], Qun Yang[3†], Jiangjing Wang[1,2†], Theodoros Dimitriadis[2], Mathias Schumacher[2], Huiru Zhang[3], Maximilian J. Müller[2], Narges Amini[4], Fan Yang[5], Alexander Schoekel[6], Julian Pries[2], Riccardo Mazzarello[7*], Matthias Wuttig[2*], Hai-Bin Yu[3*], Shuai Wei[4,8*]

Affiliations:

[1] Center for Alloy Innovation and Design (CAID), State Key Laboratory for Mechanical Behavior of Materials, Xi'an Jiaotong University, Xi'an, 710049, China.

[2] Institute of Physics (IA), RWTH Aachen University, Aachen, 52056, Germany.

[3]Wuhan National High Magnetic Field Center and School of Physics, Huazhong University of Science and Technology, Wuhan 430074, Hubei, China.

[4]Department of Chemistry, Aarhus University, 8000 Aarhus, Denmark.

[5]Institut für Materialphysik im Weltraum, Deutsches Zentrum für Luft- und Raumfahrt (DLR), 51170 Köln, Germany.

[6]Deutsches Elektronen-Synchrotron DESY, 22607 Hamburg, Germany.

[7]Department of Physics, Sapienza University of Rome, Rome, 00185 Italy.

[8]iMAT Centre for Integrated Materials Research, Aarhus University, 8000 Aarhus, Denmark.

[†] These authors contributed equally to this work.

[*] Corresponding authors. Email: shuai.wei@chem.au.dk (S.W.); haibinyu@hust.edu.cn (H.-B.Y); wuttig@physik.rwth-aachen.de (M.W.); riccardo.mazzarello@uniroma1.it (R.M.).

1. Supplementary Figures

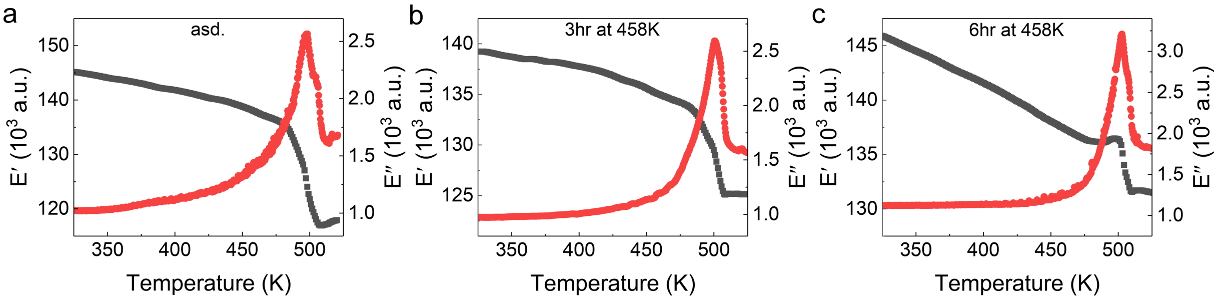

Supplementary Fig. 1. Full scans of the $Ge_{15}Sb_{85}$ powder samples using powder mechanical spectroscopy. **(a)** Storage modulus E' (black symbols) and loss modulus E″ (red symbols) of the as-deposited sample. (**b**) The annealed samples at 458 K (~0.92·T_α) for 3 hours and (**c**) for 6 hours. A constant frequency of 1 Hz and heating rate of 3 K/min were used, and a temperature calibration of -18 K is applied to all measurements.

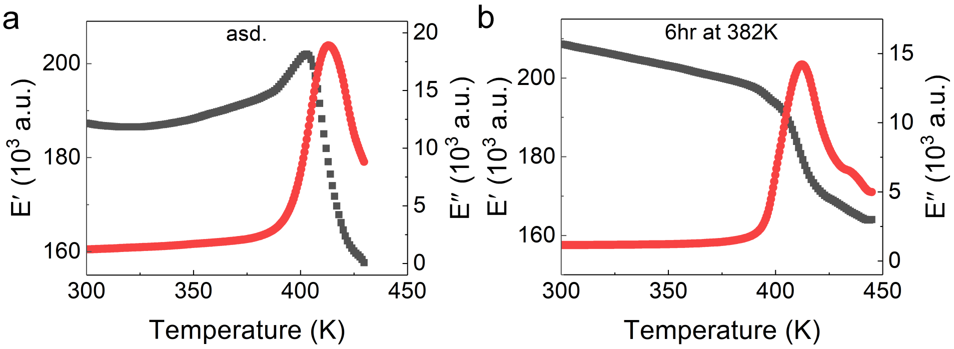

Supplementary Fig. 2. Full scans of the $Ge_{15}Te_{85}$ samples using same setting parameters as Supplementary Fig. 1. **(a)** The as-deposited; **(b)** Annealed at 382 K (~0.92·T_α) for 6 hours. Storage modulus E' and loss modulus E″ are represented by black and red symbols, respectively. A constant frequency of 1 Hz and heating rate of 3 K/min were used, and a temperature calibration of -18 K is applied to all measurements.

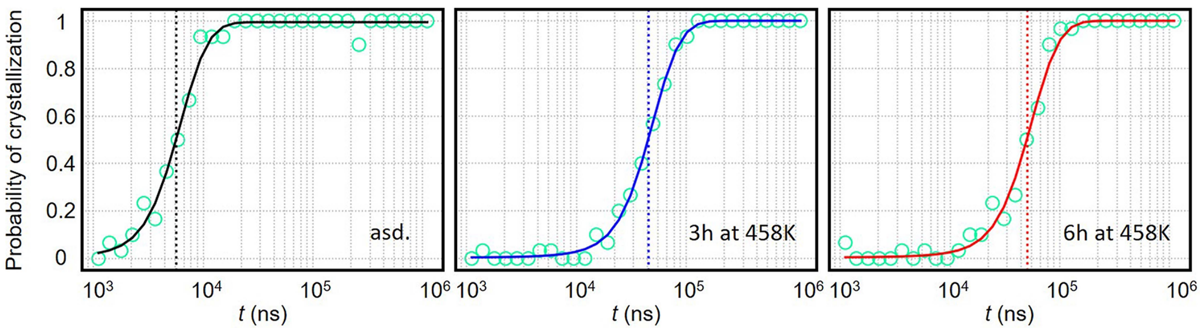

Supplementary Fig. 3. The probability of crystallization during a fine-pulse duration scan

at a constant power of 40 mW for of as-deposited, 3 hours and 6 hours annealed $Ge_{15}Sb_{85}$, respectively. The fitting for minimum crystallization time is based on the Gompertz function, which is a sigmoid function that depicts the growth mode being slowest at the start and saturated for a relatively long time. The asymptote parameter a is confined to 100 as the success rate should be no larger than 100%. The standard deviation of Y-axis (probability of crystallization), S_y, can be evaluated by the equation: $S_y = \sqrt{\frac{1}{n-1}\sum_{i=1}^{n}(y_i - \bar{y})^2}$. The calculated standard deviation of as-deposited, annealed at 185°C for 3h and 185°C for 6h films are 3.52%, 1.85% and 3.51%, respectively.

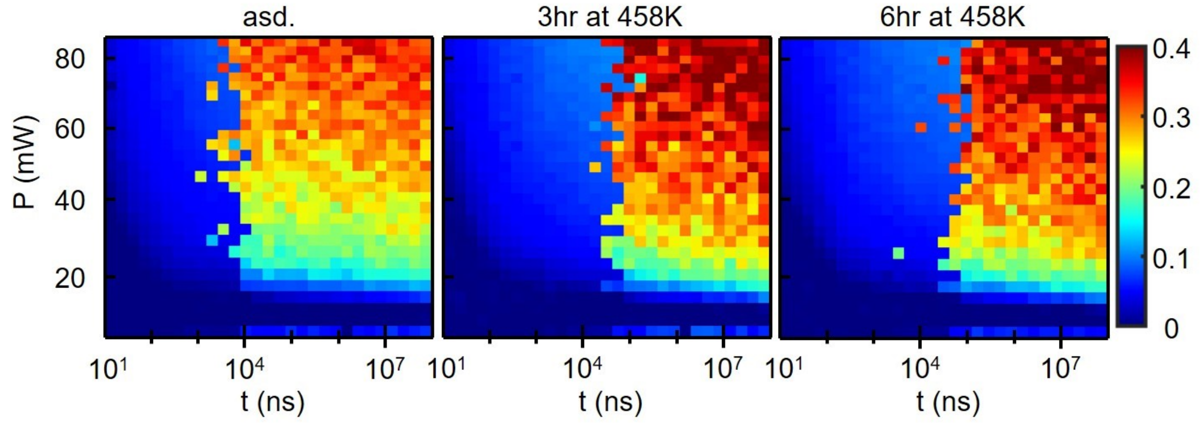

Supplementary Fig. 4. The repeated measurements of the PTE diagram. To ensure the replicability, we carried out independent measurements of amorphous $Ge_{15}Sb_{85}$ samples with a relatively low resolution (30*30 points). The results are in good agreement with the PTE diagram shown in the main text (Fig. 2a).

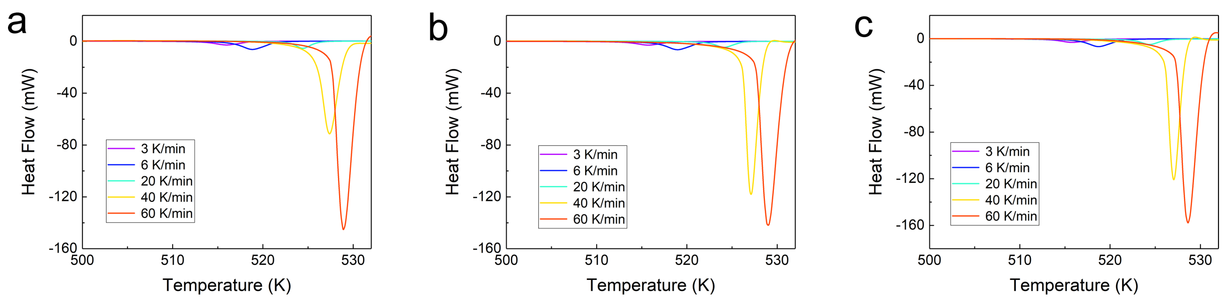

Supplementary Fig. 5. DSC scans of $Ge_{15}Sb_{85}$ at different heating rates from 3 to 60 K/min for (a) as-deposited, (b) 3hr-annealed at 458 K and (c) 6 hr-annealed at 458 K.

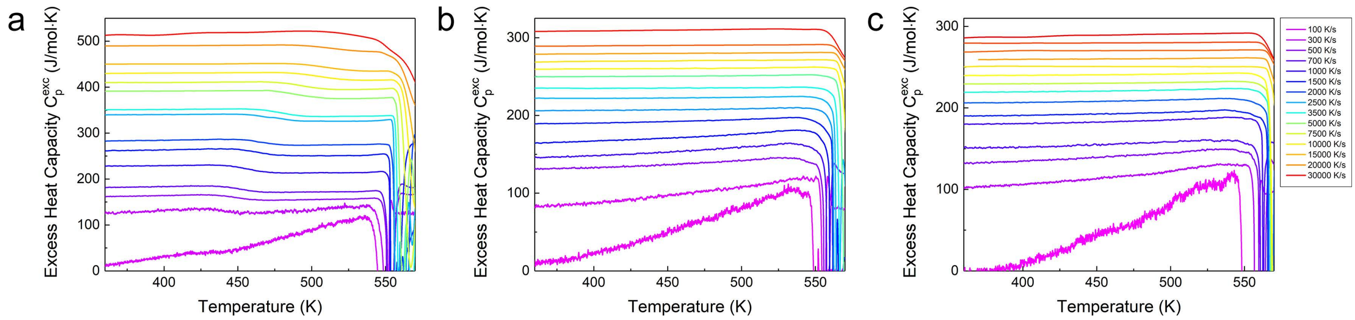

Supplementary Fig. 6. Flash DSC scans of $Ge_{15}Sb_{85}$ at the heating rates from 100 to 30000 K/s for (a) as-deposited, (b) 3hr-annealed at 458 K and (c) 6hr-annealed at 458 K. Note that the curves are vertically shifted for clarity and the lowest curve corresponds to 100 K/s with a lower signal-to-noise ratio.

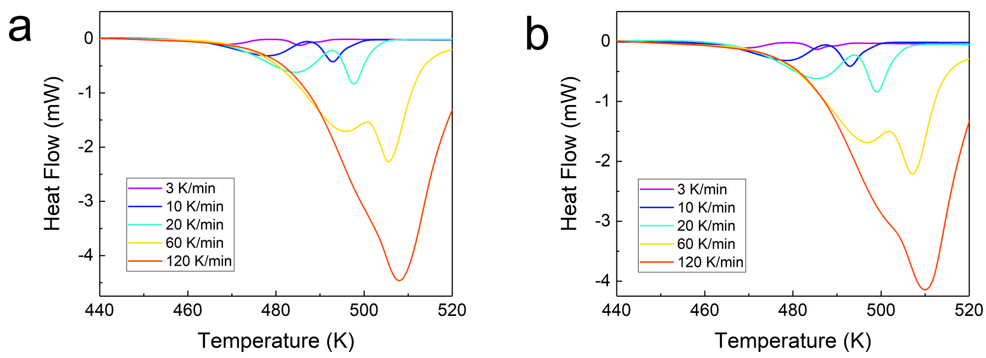

Supplementary Fig. 7. DSC scans at different heating rate for (a) as-deposited and (b) 6hr-annealed $Ge_{15}Te_{85}$.

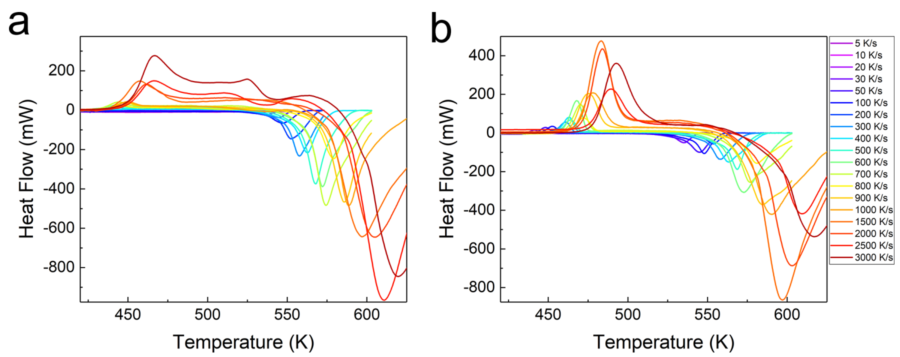

Supplementary Fig. 8. Flash DSC scans at higher heating rates for (a) as-deposited and (b) 6hr-annealed $Ge_{15}Te_{85}$. Note that due to the low melting point, a heating rate above 3000 K/s shifts the T_p into the melt, partially bypassing the crystallization.

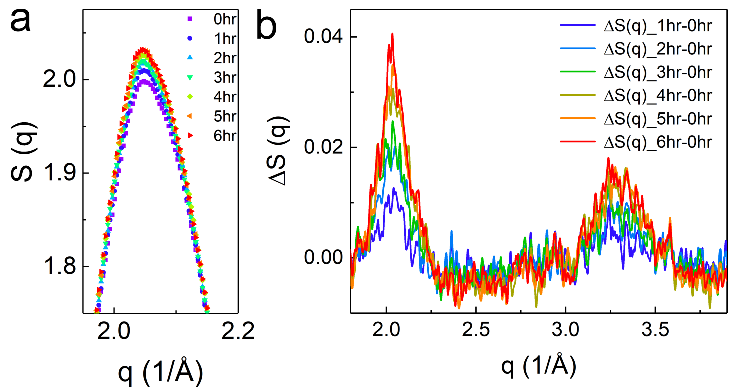

Supplementary Fig. 9. (a) The zoom-in of the first *S(q)* peaks of $Ge_{15}Sb_{85}$ upon isothermal annealing at 458 K for 6 hours. (b) The difference plot ΔS(q)=S(q,t)-S(q,t=0hr). The trend of the peak sharpening is observed upon annealing.

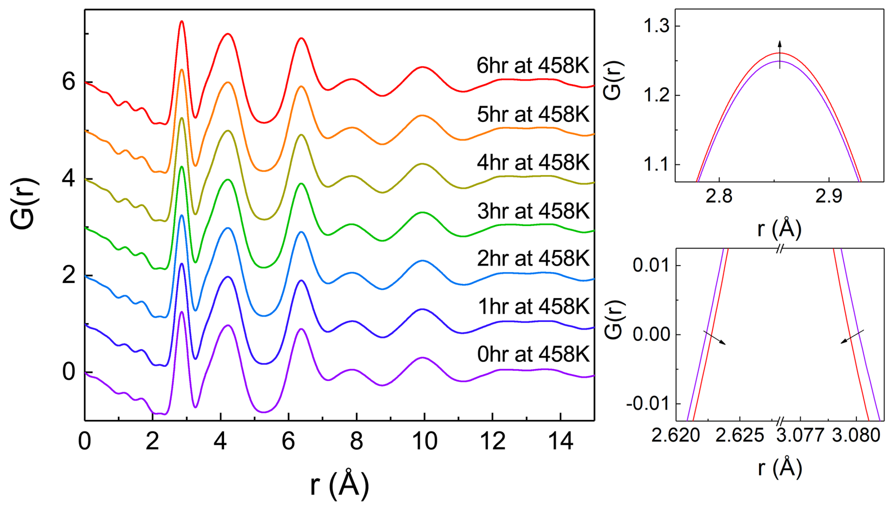

Supplementary Fig. 10. The reduced pair distribution functions *G(r)* during the isothermal annealing of the amorphous $Ge_{15}Sb_{85}$ at 458 K up to 6 hours. The spectra are vertically shifted for an easy view. Inset: the zoom-in of the peak intensity and full-width-half-maximum (FWHM) of the first main peak of *G(r)*. Only the 0hr-annealed and 6hr-annealed curves are shown in the insets.

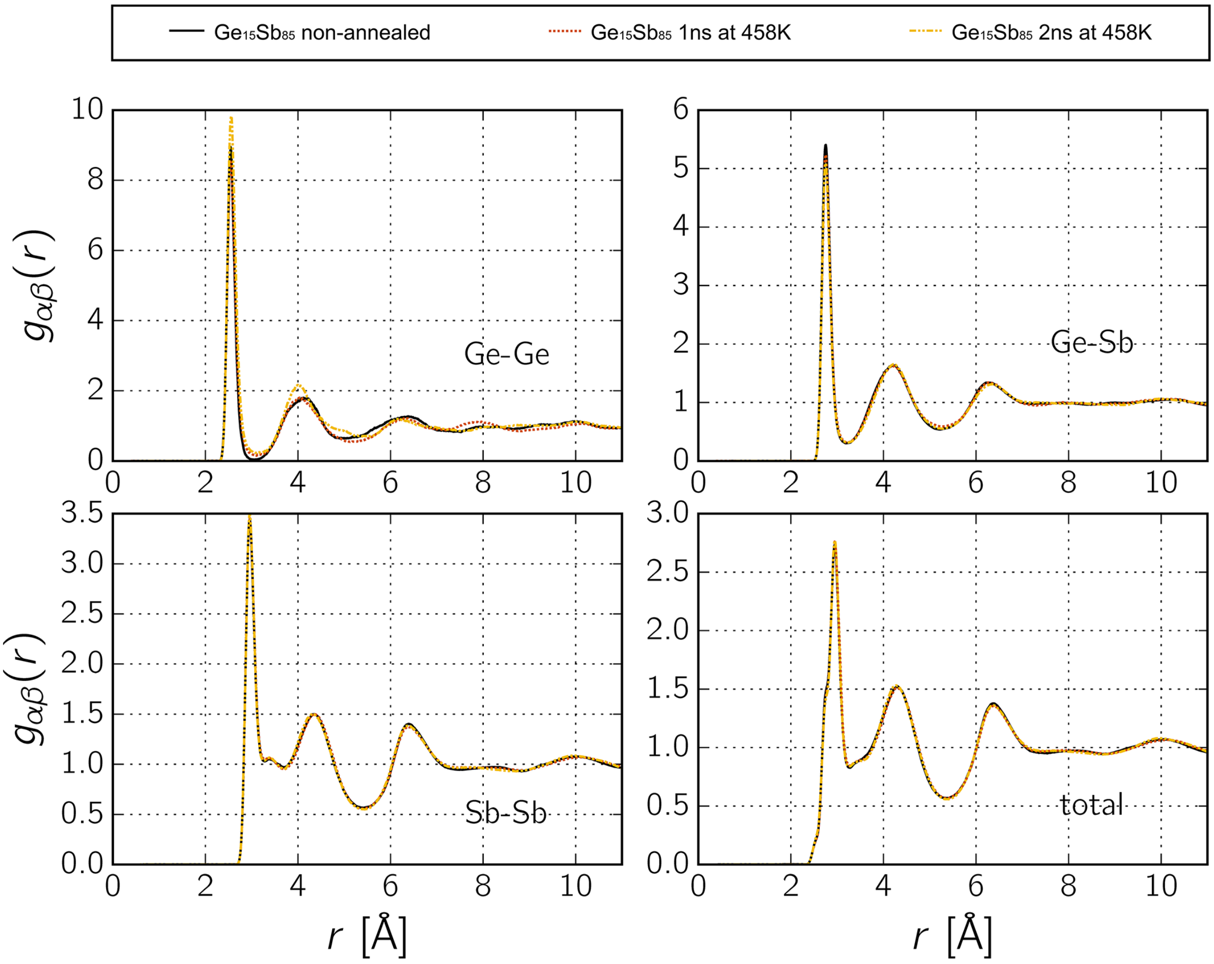

Supplementary Fig. 11. The calculated partial and total pair distributions of amorphous $Ge_{15}Sb_{85}$. The plots are obtained from AIMD simulations employing the PBE exchange correlation function. Plotted for a direct comparison are the total and partial *G(r)* of the non-annealed sample, of a sample kept at ~458 K for 1 ns and 2 ns before re-quenched to ~285 K.

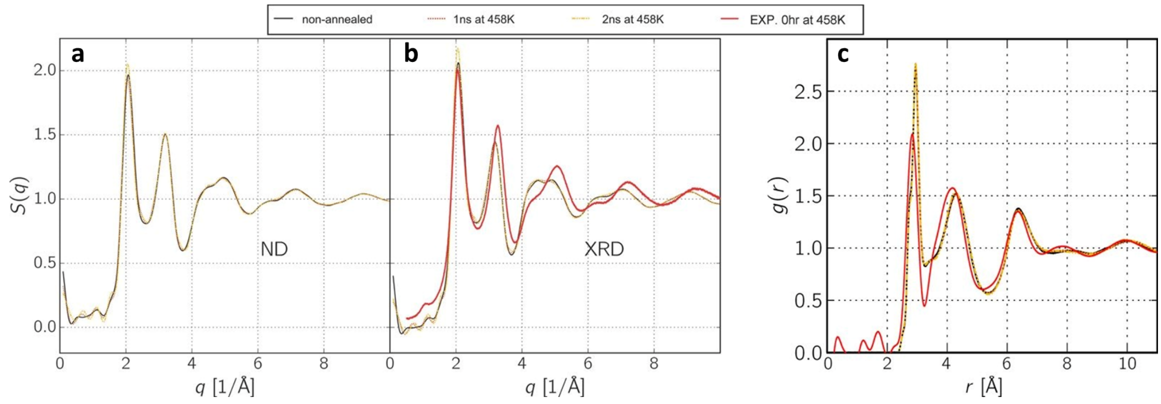

Supplementary Fig. 12. (**a**) Simulated *S(q)* of $Ge_{15}Sb_{85}$ for neutron scattering (ND). (**b**) Simulated *S(q)* of $Ge_{15}Sb_{85}$ for X-ray scattering (XRD) compared with the experimental XRD data (red curve). The agreement is satisfactory for a qualitative interpretation, as all the main features are reproduced in the simulation. (**c**) Simulated *g(r)* functions are compared with the

experimental *g(r)* (red curve) converted from *G(r)*. Note that the number density ρ_0 upon annealing at 458 K is needed to convert *G(r)* to *g(r)* via $g(r)=G(r)/4\pi\rho_0+1$. Unfortunately, we do not have this density information and thus assumed ρ_0 is roughly equal to the value of room-temperature density. The level of agreement is in line with previous computational work on PCMs employing standard PBE functionals.

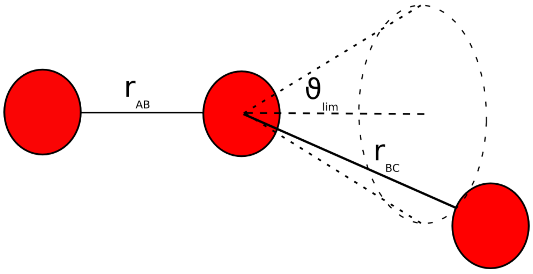

Supplementary Fig. 13. The analysis of angular limited three-body correlation (ALTBC) concerns triples of atoms. Within a given maximum angle θ_{lim} between two inter-particle vectors $\boldsymbol{r_{AB}}$ and $\boldsymbol{r_{BC}}$, the statistics of two inter-particle distances r_{AB} and r_{BC} will be determined. Thus, the 2D distribution (contour plot) of (r_{AB}, r_{BC})-values indicates the chain-like three-body correlations. If the peaks in the contour plot are *off* the diagonal $r_{AB} = r_{BC}$, it indicates a chain of alternating short and long bonds and Peierls-like distortions (*73*).

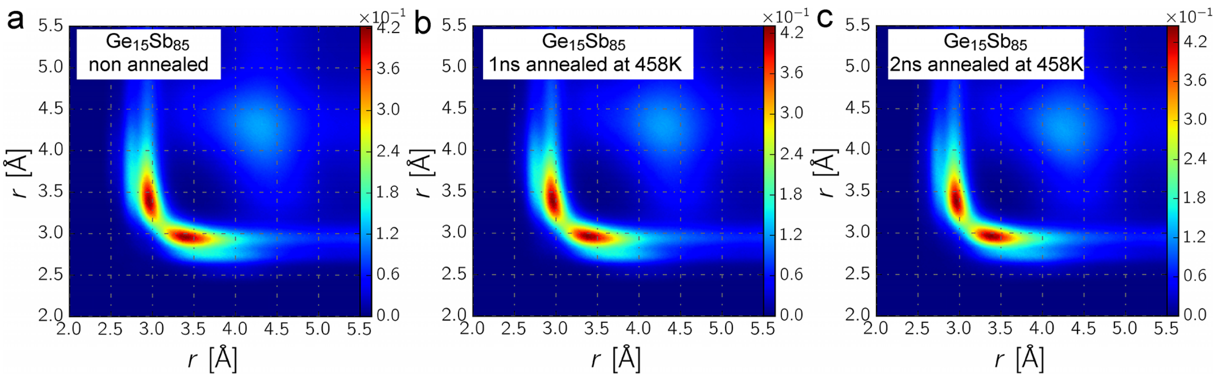

Supplementary Fig. 14. Total ALTBC functions of amorphous $Ge_{15}Sb_{85}$ at ~285 K. (a) Non-annealed. **(b)** After annealing at ~458 K for 1 ns. **(c)** After annealing at ~458 K for 2 ns.

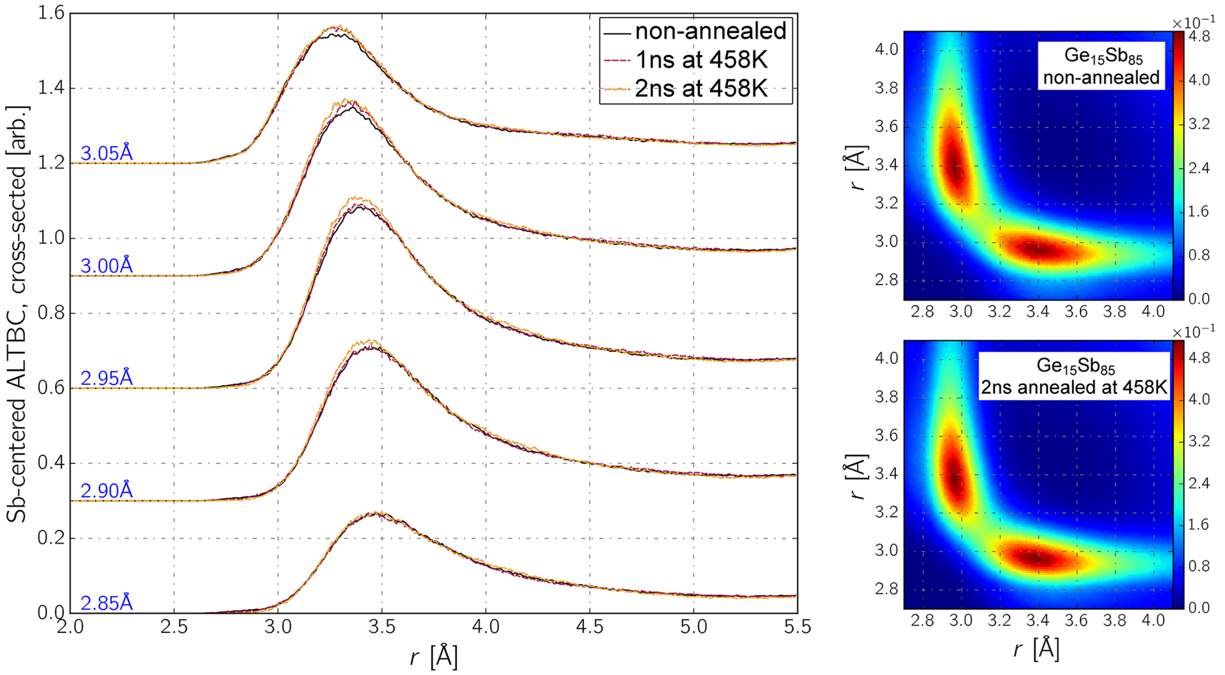

Supplementary Fig. 15. The cross-section plots along *r*=2.85-3.05 Å of the Sb-centered ALTBC. Three $Ge_{15}Sb_{85}$ samples are included, i.e., the melt-quenched sample and samples annealed at ~458 K for 1 ns and 2ns before re-quenched to ~285 K. The 2D ALTBC contours of two samples are also given here. Note that the increase in peak height in cross-section plots is consistent with the trend in total ALTBC; therefore, the Sb-centered contribution dominates the total ALTBC due to its high atomic percentage in the alloy.

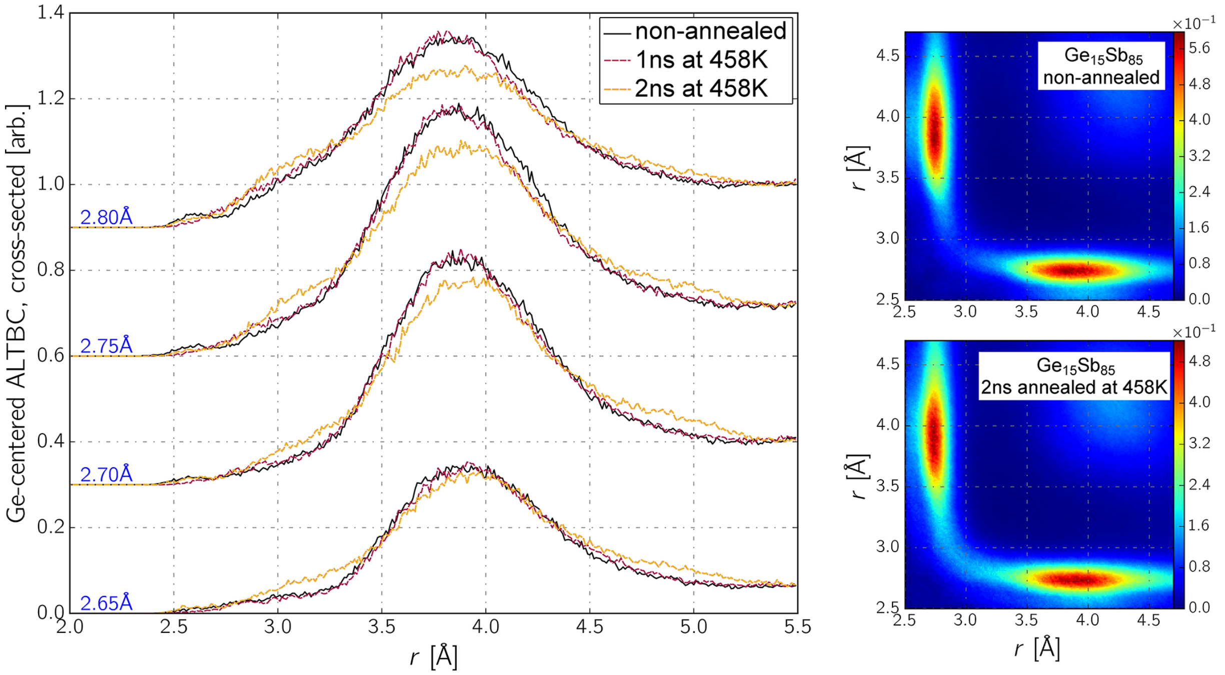

Supplementary Fig. 16. The cross-section plots along *r*=2.85-3.05 Å of the Ge-centered ALTBC. Three $Ge_{15}Sb_{85}$ samples are included, i.e., the melt-quenched sample and samples annealed at ~458 K for 1 ns and 2 ns before re-quenched to ~285 K. The 2D ALTBC contours of two samples are also given. The change in peak height does not show a clear trend as

observed in total ALTBC. Thus, Ge-centered ALTBC does not appear to dominate the total ALTBC.

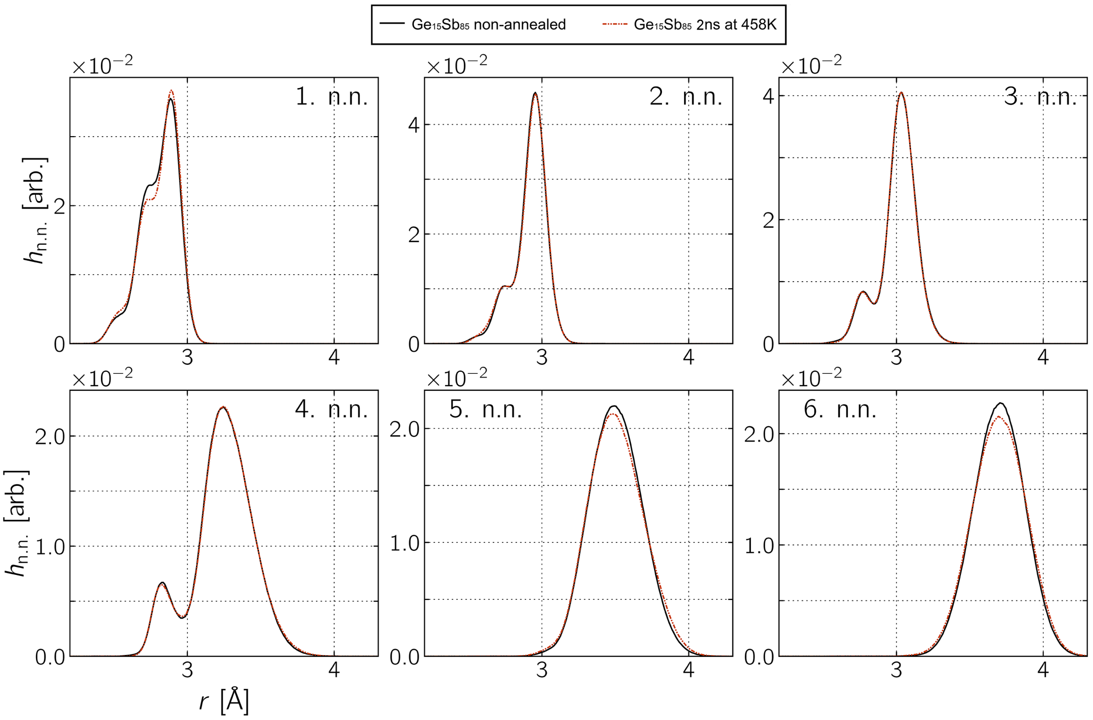

Supplementary Fig. 17. Total nearest-neighbor distribution of amorphous $Ge_{15}Sb_{85}$. The centers of the histograms shift to higher distances with increasing nearest neighbor order, and the overlaps between histograms of different nearest neighbor orders are also obvious. The peaks of the orders 1, 2, 3, and 4 show a slight but clear enhancement to higher intensity, which is dominated by Sb-centered nearest neighbor distribution, see Supplementary Fig. 18-21. On the contrary, fifth and sixth order peaks reveal weaker values after annealing.

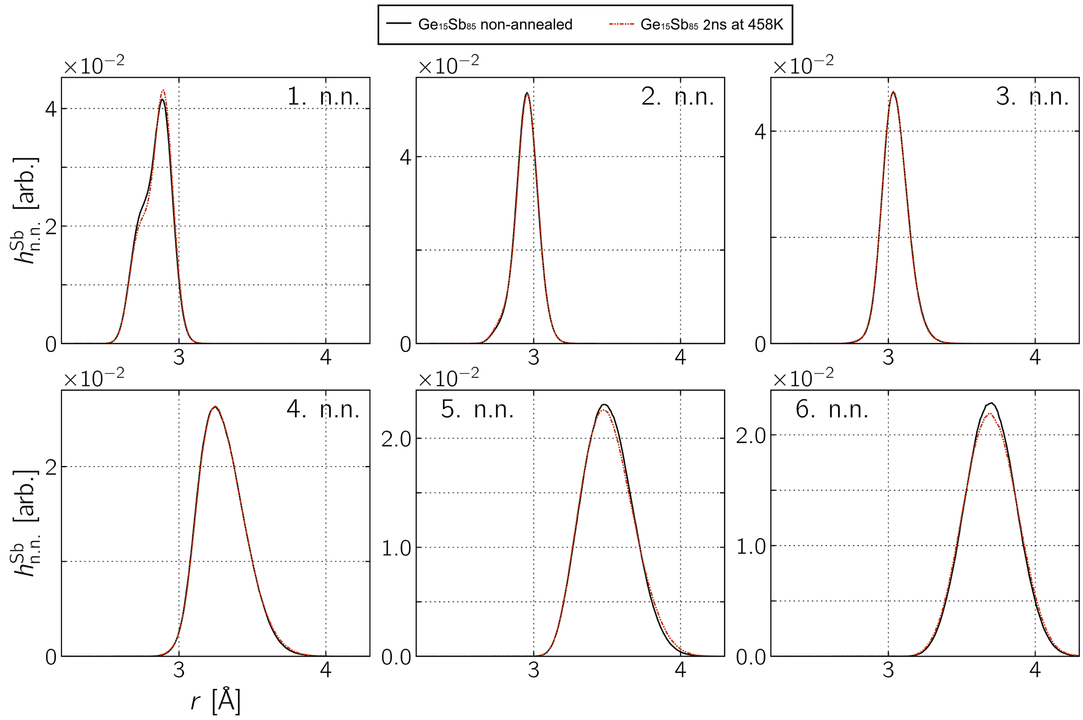

Supplementary Fig. 18. Sb-centered nearest-neighbor distribution of amorphous $Ge_{15}Sb_{85}$.

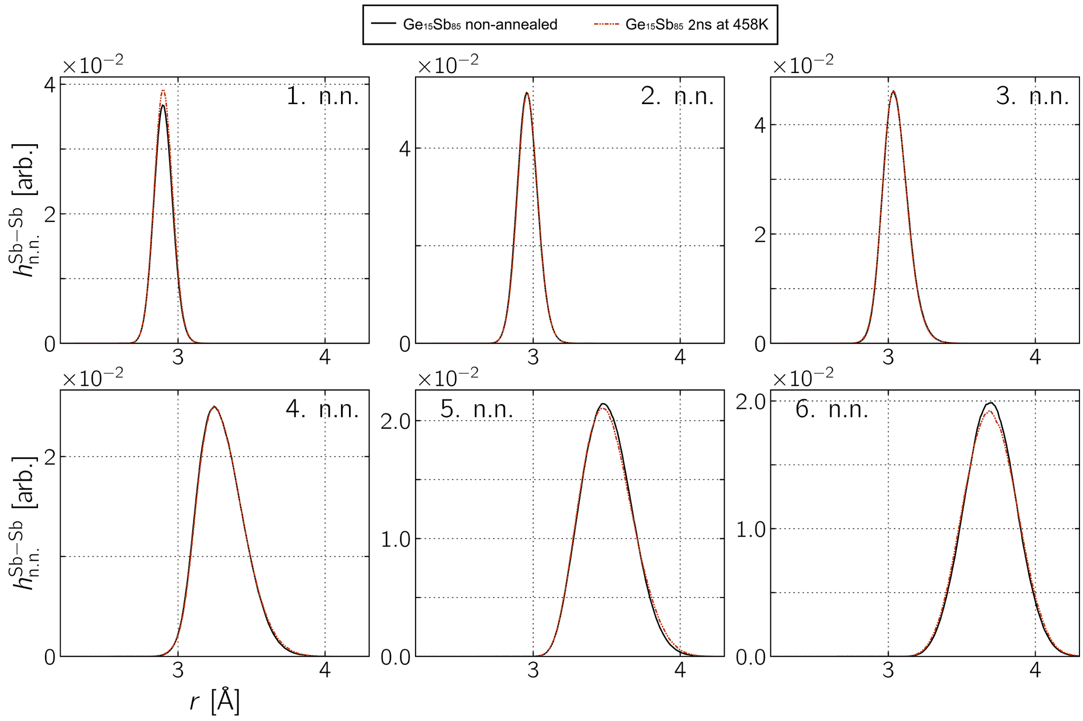

Supplementary Fig. 19. Sb-Sb nearest-neighbor distribution of amorphous $Ge_{15}Sb_{85}$.

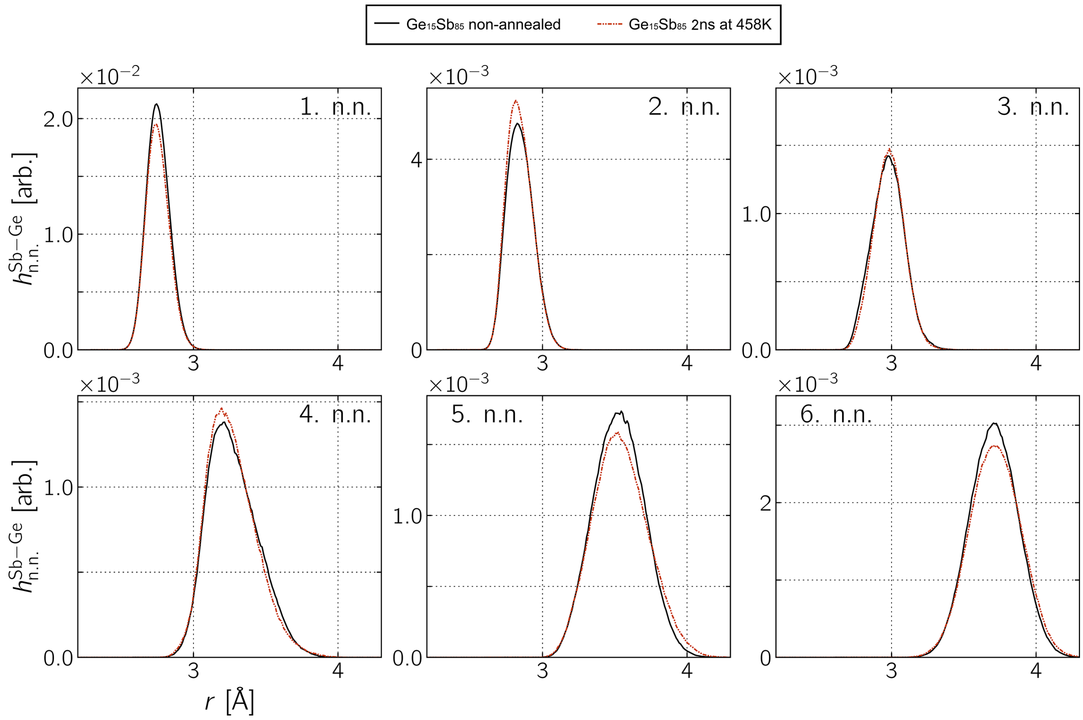

Supplementary Fig. 20. Sb-Ge nearest-neighbor distribution of amorphous $Ge_{15}Sb_{85}$.

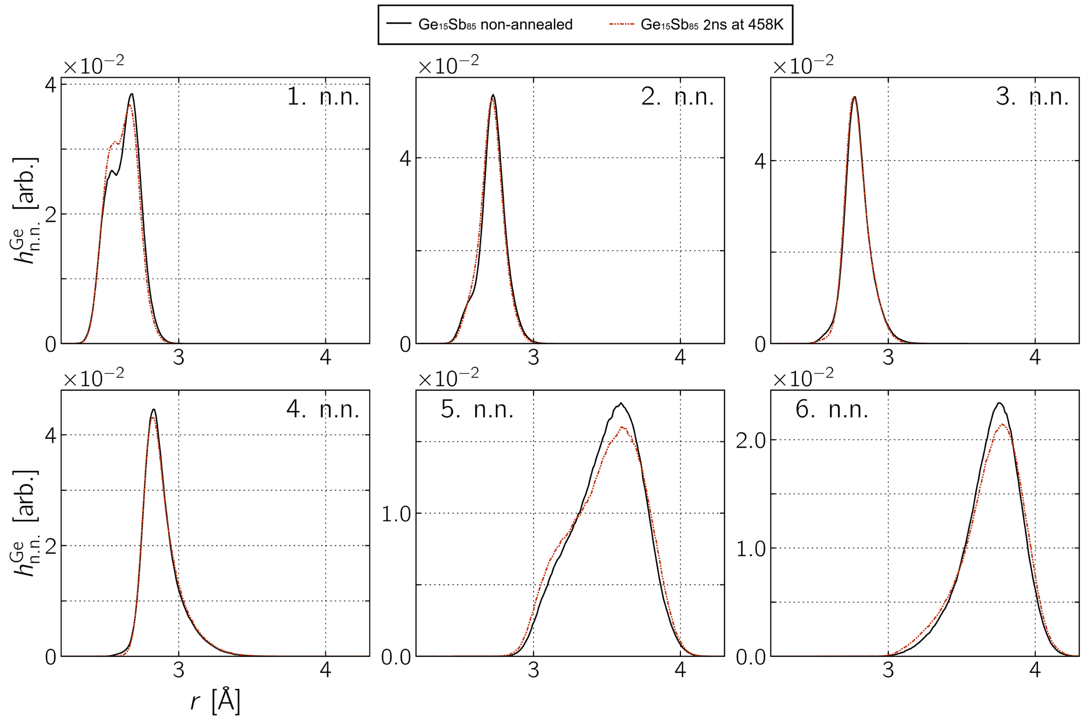

Supplementary Fig. 21. Ge-centered nearest-neighbor distribution of amorphous $Ge_{15}Sb_{85}$.

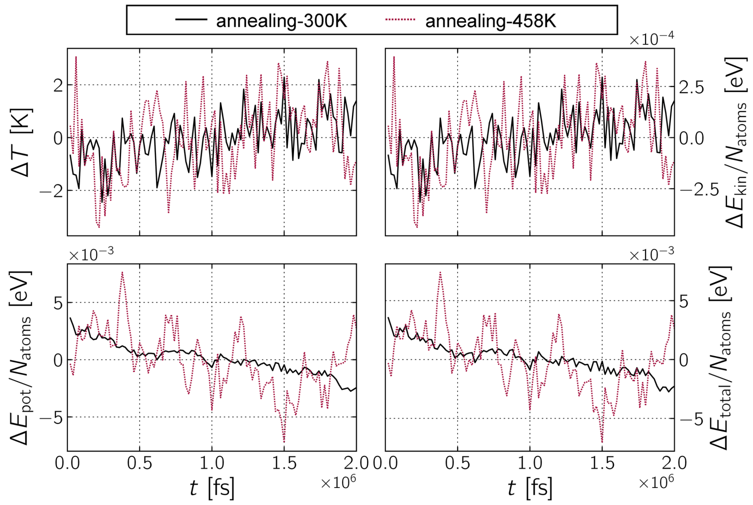

Supplementary Fig. 22. The drift of the potential and total energy along the isothermal annealing at 300 K and 458 K for 2 ns. At 300 K, the drift is of the order of $4{\cdot}10^{-3}$ eV/ns per atom. The similar trend in the drift appears at 458 K despite a larger uncertainty. The drifts stem from genuine relaxation of the amorphous state.

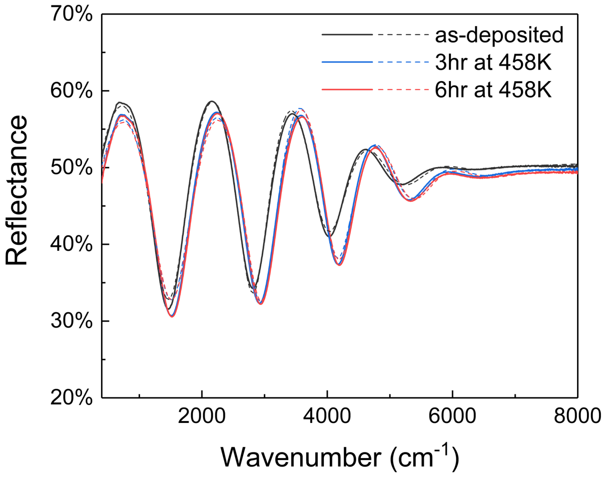

Supplementary Fig. 23. Reflectance spectra of amorphous $Ge_{15}Sb_{85}$ as measured by FTIR at room temperature. Black solid line is as-deposited $Ge_{15}Sb_{85}$, while blue and red solid lines are amorphous $Ge_{15}Sb_{85}$ annealed at 458 K for 3 and 6 hours, respectively. The corresponding dashed lines represent the fitting results employing Tauc-Lorentz model.

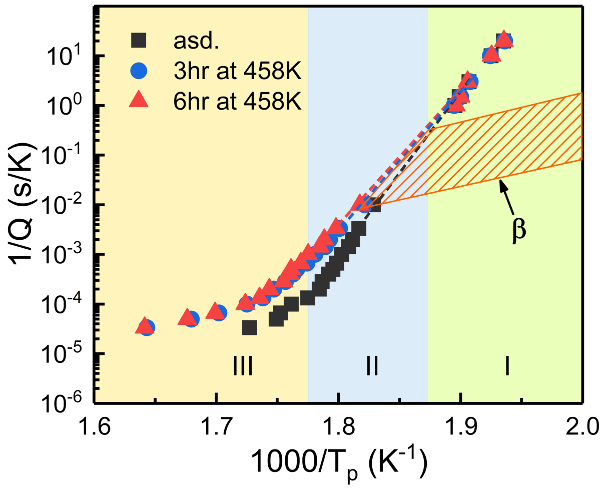

Supplementary Fig. 24. Illustration for the suppression of β-relaxation and its influence on crystallization kinetics in $Ge_{15}Sb_{85}$. The shadow region represents the upper and lower boundary of T_β as a function of Q. T_β crosses T_p at the heating rate around which the effect of suppressing β-relaxation starts to show up in crystallization.

2. Supplementary Table

Supplementary Table S1 Standard deviations of T_p in the FDSC measurements for as-deposited (SD_{asd}), 3 hours annealed (SD_{3hr}) and 6 (SD_{6hr}) hours annealed $Ge_{15}Sb_{85}$ samples. The standard deviation values are calculated from the average of 10 measurements for each heating rate.

Heating rate Q (K/s)	1E2	3E2	5E2	7E2	1E3	1.5E3	2E3	2.5E3	3.5E3	5E3	7.5E3	1E4	1.5E4	2E4	3E4
SD _asd(K)	0.7	0.9	1.7	1.1	0.9	1.8	2.0	1.6	1.6	1.7	1.8	2.1	1.9	5.0	3.3
SD_3hr (K)	1.2	1.9	1.2	0.6	1.0	2.4	2.3	1.0	1.1	1.7	2.6	6.4	3.1	3.5	7.7
SD_6hr (K)	0.4	1.3	1.0	1.2	1.1	1.4	1.4	1.6	1.3	2.6	3.0	2.7	5.8	6.7	4.2

3. Supplementary Notes

The timescales of β-relaxation and crystallization

At the low heat rates Q~1-50 K/s, which is within the typical range of conventional DSC, the suppression of the β-relaxation has little impact on the T_p values. In this regime, the temperature of the β-relaxation T_β occurs well below that of the crystallization. Both occur at the relatively low temperatures, where the overall dynamics are slow. Although the atomic mobility in some local regions, corresponding to the β-relaxation, is higher than that of the

slow α-processes in the surrounding matrix, it is apparently not sufficient to affect the crystallization behavior, probably because the overall mobility is still not enough for nucleation and growth of crystals at this temperature. Therefore, the vanishing of β-relaxation with low heating rates barely causes any changes in T_p.

With the increasing heating rate, both T_β and T_p are shifted to higher temperatures. However, their heating-rate dependences differ due to their different activation energies. Using the data in Fig. 1a, we can estimate the peak temperature of β-relaxations T_β as a function of the heating rate Q. According to Ref. (*25*), T_β is related to the testing frequency f of DMA (PMS in our case) measurement *via* the equation $f = f_0 \cdot exp(-E_\beta/RT_\beta)$, where E_β is the activation energy of β-relaxations, R is the gas constant, and f_0 is a pre-factor. It has been shown that E_β can be expressed by an universal relation with the standard T_g (T_g at 20 K/min in a DSC measurement) of the materials, $E_\beta = (26\pm2)\cdot RT_g$ where the pre-factor 26 is a fitted parameter for a range of glassy materials (e.g., metallic, polymeric, molecular and oxide glasses) (*74, 75*). As demonstrated in Ref. (*76*), the testing frequency f and the heating rate Q are linked by a linear relation $n_s \cdot f = Q$, where n_s is the shift factor n_s. As shown in our previous work, after temperature calibration, T_α measured at 1 Hz in PMS is approximately equal to the standard T_g measured in DSC with a heating rate of Q = 20 K/min. This results in a n_s of 1/3 K. If we assume $T_g = T_\alpha$ = 500 K and T_β is located in the range from 440 K to 490 K at the frequency of 1 Hz, with a known shift factor n_s between the PMS frequency and the heating rate, we can identify the pre-factor f_0 (6.78×10^{12} Hz when T_β = 440 K; 3.33×10^{11} Hz when T_β = 490 K), E_β (108 kJ/mol) and finally the relationship between T_β and the heating rate Q, i.e. $Q = n_s \times f_0 \times \exp\left(-\frac{E_\beta}{R\times T_\beta}\right) = \frac{1}{3} \times 6.78 \times 10^{12} \times \exp\left(-\frac{1.08\times10^5}{8.314\times T_\beta}\right)$ if we assume T_β = 440 K. Note the value of f_0 will be different if a different T_β is used. The estimated range of T_β at different heating rates is sketched by the shadow region in Supplementary Fig. 24, together with the crystallization peak temperature T_p. Note the determination of T_β at 1 Hz is not trivial, as the peak of β-relaxation has partially merged into the α-relaxation peak due to the coupling of the two relaxation processes. Therefore, the range of T_β at 1 Hz is estimated using an upper and a lower boundary temperature, 440 K < T_β < 490 K. As shown in Supplementary Fig. 24, the T_β is shifted to higher temperatures with larger heating rates and crosses T_p in the range of ~530 K-550 K at the heating rate of about 50 to 10^2 K/s. At this crossover temperature, the timescales of the crystallization and the β-relaxation become comparable. As shown in Fig. 3c, at almost the same rate, the effect of thermal annealing on crystallization becomes pronounced. This is hardly a coincidence and implies that β-relaxation starts to dominate the crystallization when the timescales of both processes are comparable.
